# Spectrum-Efficient Resource Allocation in Multi-Radio Multi-Hop Cognitive Radio Networks

**DOI:** 10.3390/s19204493

**Published:** 2019-10-16

**Authors:** Bin Han, Ying Luo, Min Zeng, Hong Jiang

**Affiliations:** Network and Communication Research Institute, College of Information Engineering, SouthWest University of Science and Technology, Mianyang 621010, Sichuan Province, China; hanbin@swust.edu.cn (B.H.); zengm@swust.edu.cn (M.Z.); jianghong@swust.edu.cn (H.J.)

**Keywords:** multi-hop cognitive radio, spectrum allocation, power allocation, spectral efficiency

## Abstract

The multi-hop cognitive radio network (CRN) has attracted much attention in industry and academia because of its seamless wireless coverage by forming multi-hop links and high spectrum utilization of cognitive radio (CR) technology. Using multi-slot statistical spectrum status information (SSI), this work investigates the average spectrum efficiency (SE) of a multi-radio multi-hop (MRMH) CRN where each hop is permitted to use different spectra and long-distance hops can reuse the same idle primary user (PU) spectrum. Faced with the modeled SE problem, which is a complex non-convex fractional mixed integer nonlinear programming (MINLP) problem, the optimal spectrum and power allocation for multi-hop links in multi-slot and multi-channel scenarios can be obtained with the proposed successive multi-step convex approximation scheme (SMCA). As shown through computational complexity and simulation analysis, SMCA can obtain an approximate lower bound of the optimal solution for the modeled SE problem with a lower computational cost. Furthermore, some potential relationships between network performance and spectrum idle rate can be easily discussed with SMCA, which can provide some sensible deployment strategies for the MRMH CRN in future multi-slot scenarios.

## 1. Introduction

The wireless spectrum is the cornerstone of wireless communication. In recent years, the spectrum has become scattered or unavailable, and especially the licensed spectrum. There are two reasonable aspects to explain this phenomenon. In the first place, the continuous growth of wireless access terminals leads to severe shortages and competition for spectrum resources [1,2]. Secondly, the fixed spectrum allocation mechanism of the licensed spectrum further results in poor spectrum utilization, which is called spectrum under-utilization [3,4]. As reported by the Federal Communication Commission, on a daily basis, the waste rate of a dispatched spectrum may be as high as 88% [5]. Faced with the above obstacles, cognitive radio as a dynamic spectrum access mechanism is being proposed as a key possible technology to address the issues of spectrum scarcity and under-utilization [6].

Cognitive radio (CR) technology allows the secondary user (SU) to transmit using the idle time of the spectrum licensed to the primary user (PU). In this way, CR can efficiently improve spectrum utilization. Consequently, a wireless network where SU and PU coexist at the same time is referred to as a cognitive radio network (CRN) [7]. Apparently, to realize cognitive radio networks (CRNs), spectrum sensing and spectrum management are two important building blocks that have been extensively studied in single-hop CRNs. However, in the last few years, researchers have gained a great deal of interest in investigating the multi-hop CRN [8,9,10,11,12,13,14,15,16,17,18]. The major reason is that wireless nodes can communicate with each other over long distances by forming multi-hop links [8]. In other words, the multi-hops can achieve a seamless wireless communication network, which deserves a clear and more detailed study. Nevertheless, the successful implementation of a multi-hop CRN requires a clear recognition of the internal distinction between single-hop and multi-hop CRNs. Thus, as stated in [9], the crucial technical difference between single-hop and multi-hop CRNs comes from two aspects: routing and spectrum allocation.

In keeping with the original intention of CR, this work allows multiple cognitive hops to use a spectrum at the same time. For instance, when the distance between two hops reaches a respectful distance, the mutual interference can be tolerated. In this way, spectrum utilization can be enhanced by sacrificing a certain amount of power to endure the mutual interference. Based on the above assumption, this work concentrates on designing a reasonable and efficient spectrum allocation and power control scheme to improve the spectrum utilization (i.e., spectral efficiency: SE) of multi-hop CRNs. Accordingly, the contributions of this work can be summarized as follows:

### 1.1. Contributions

- Firstly, with reference to the statistical spectrum status information (SSI), an average SE programming problem of a multi-hop cognitive link is modeled to address the spectrum and power allocation simultaneously.
- Subsequently, faced with the complex non-convex fractional mixed integer nonlinear programming (MINLP) problem, we propose a successive multi-step convex approximation scheme (SMCA) to solve it.
- Thirdly, the complexity and simulation analysis show that SMCA can not only obtain a sub-optimal SE for multi-hop CRN but also has a lower computational complexity. In addition, the network performance is analyzed along with the change of the spectrum idle rate, which can give us some potential deployment references for multi-hop CRN under multi-slot scenarios in the near future.

### 1.2. Related Works

The multi-hop CRN has become an attractive research topic in industry and academia due to the inherent demand for high spectrum utilization, long-distance full-coverage wireless communication. However, how to reasonably choose the relays from the cognitive multi-hop source to the destination and assign appropriate channel resources to support multi-hop communication are two main research interests in multi-hop CRN.

In view of the proper relay-choosing schemes for cognitive multi-hop source–destination paths, or cognitive multi-hop routing approaches, most scholars are inclined to establish the multi-hop CRN as a graph theory model [10,11,12], which is also a common approach in multi-channel ad hoc networks. But, by comparing with the intrinsic nature of ad hoc networks, more consideration should be given to spectrum resource allocation when we design the resources assignment of the multi-hop CRN [13]. In cognitive radio ad-hoc networks, [19] proposes an optimal resource allocation strategy by constructing a Stackelberg game between the PU and SUs, and a non-cooperative game among SUs, and obtains the optimal equilibria including the power of every SU and spectrum leasing time in relay task. But this scheme is only applicable to cognitive networks where the primary user and the secondary user have a leasing relationship. In cognitive radio networks, [20] proposes a fully distributed game-theory-based algorithm to achieve optimal power control and relay selection under the constraint of QoS requirements of the primary user and secondary user, with the aim of maximizing the individual capacities of the secondary links. However, this algorithm does not discuss the spectral efficiency maximization of the system. Thus, as mentioned in [13], the diversity of CR, such as the dynamic availability of the spectrum, the wide range and heterogeneity of radio frequencies, and the dynamically changing topology and incomplete radio information, should be given more attention. Hence, this work is more concerned with the spectrum resource allocation of multi-hop CRNs.

**Figure 1:**
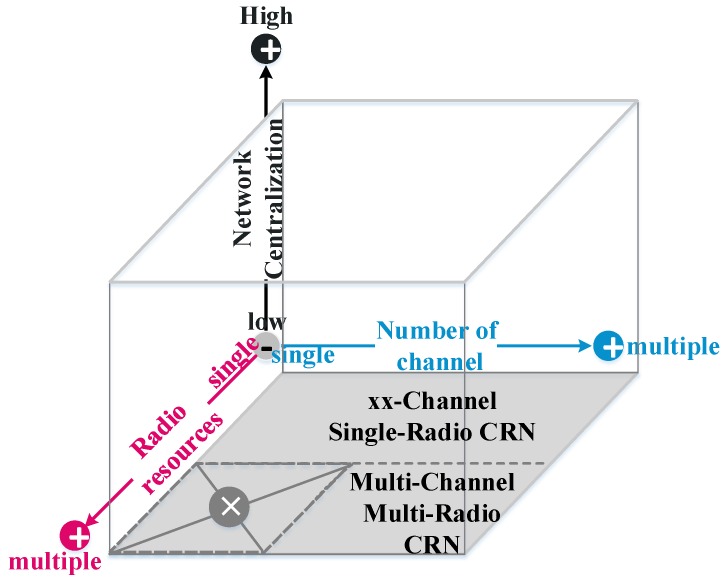
The spectrum assignment of the multi-hop cognitive radio network (CRN) segmented into four parts across three dimensions.

From the above discussion, and as demonstrated by Figure 1, the spectrum assignment of multi-hop CRNs can be segmented into four major parts according to three dimensions: number of channels (i.e., spectrum resources), radio resources, and network centralization.

The spectrum usage rule of multi-hop CRNs is based on the current available number of channels or spectra and the radio resources equipped by cognitive terminals. From the perspective of radio resources, multi-hop CRNs can be divided into multi- and single-radio scenarios. In the single-radio multi-hop (SRMH) CRN, a multi-hop link transmits data over a suitable channel using a single-radio resource. Under SRMH CRNs, the transmission of the cognitive hops can be achieved by multiplexing the PU’s spectrum resource through the reasonable power control scheme (i.e., underlay mode) [14] or by using the idle time of PU’s spectrum resource through the time division multiplexing scheduling between multiple hops (i.e., overlay mode) [15,16]. Regardless of the overlay or underlay mode, studies of SRMH CRNs must allocate the spectrum occupancy time for each hop. However, there are two significant deficiencies in SRMH CRNs. Initially, the transmission congestion may occur at some central nodes of the multi-hop link. At this moment, the single-radio scenario may cause a large transmission delay and, more seriously, transmission failure. What’s more, in the overlay mode, it is clear that the multi-hop cognitive link must vacate the current spectrum when the licensed PU is active. At this point, the transmission of multi-hop link will be interrupted. It means that the multi-hop routing is not robust due to the spectrum usage scheme. Therefore, to handle the above two shortcomings of SRMH CRNs, multi-radio resources are equipped with transmission terminals.

In multi-radio multi-hop (MRMH) CRNs, each hop of the multi-hop link can transmit or receive at the same time under different spectra. This makes it possible for multi-channel to be the basic condition and is an important guarantee for implementing MRMH CRNs. In this way, the transmission congestion occurring at the central node of the multi-hop link can be alleviated. Accordingly, Tang et al., studied a joint resource allocation scheme including rate matching, channel assignment, and routing in MRMH CRNs [17]. Moreover, it is well known that in the overlay mode, the performance of the SU is closely related to the licensed spectrum vacancy time of the PU [16]. To decrease the transmission interruption that comes from the sudden activation of a single spectrum resource, and finally improve the performance stability of the multi-hop link, multiple spectrum resources can be assigned to the transmission or receiving of each hop [10,11,18]. In this way, multi-radio can provide a robust routing for multi-hop CRNs. Consequently, the current research directions in MRMH CRNs are appropriate spectrum assignment under the robustness constraint of the multi-hop link [11] or transmitting data over the minimal number of hops [18].

As mentioned above, the MRMH CRN can not only increase routing robustness but also can alleviate transmission congestion by sacrificing some spectrum resources. However, with the full consideration of the original purpose of CR, how to promote SE in MRMH CRNs is a worthy research topic. Hence, this work assumes that each hop is permitted to use a different spectrum, and hops with long distances can reuse the same spectrum at the same vacant time of the PU. Moreover, similar to [11], under the statistical SSI, the average SE of a multi-hop link for MRMH CRNs is investigated and the optimal spectrum and power allocation in a multi-time and multi-channel scenario can be obtained simultaneously.

### 1.3. Overview of the Sections

The rest of this work is organized as follows. The system model is described in Section 2. Then, the average SE maximization problem for the cognitive multi-hop link is formulated in Section 3. In Section 4, a successive multi-step convex approximation scheme is proposed. The theoretical computational complexity and simulation analysis of the proposed algorithm are discussed in Section 5. Finally, we draw a conclusion in Section 6.

## 2. System Model

**Figure 2:**
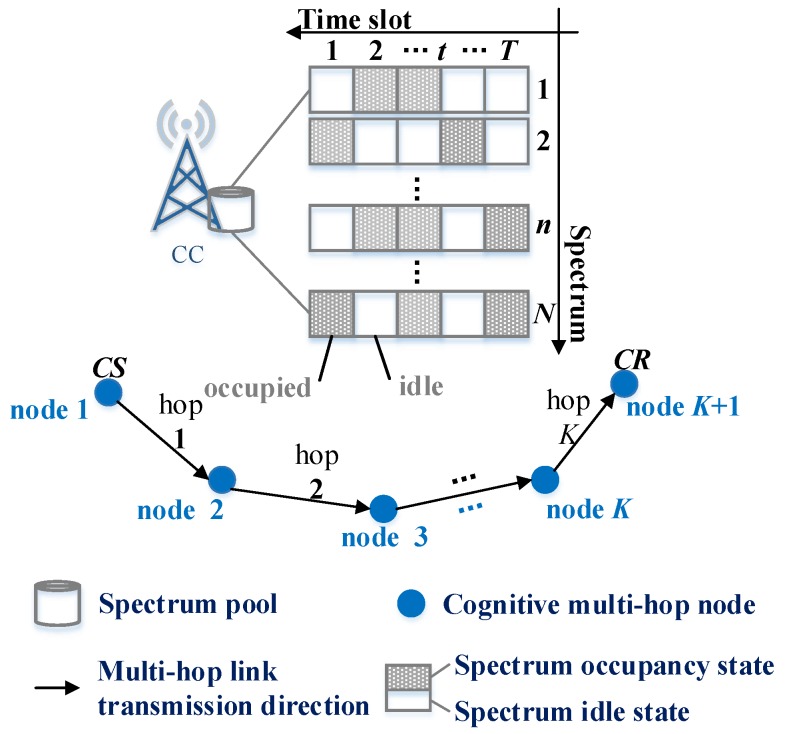
System description of the cognitive multi-hop multi-channel transmission model.

Considering the cognitive multi-channel multi-hop (MCMH) scenario described by Figure 2, the cognitive sender (CS) can communicate with the long-range cognitive receiver (CR) over a *K*-hop transmission link. For simplicity, the transmitting node and receiving node of the *k*-th hop are numbered *k* and k+1, respectively. The cognitive users are only permitted to transmit when the status of the spectrum assigned to primary users is idle. Thus, the core controller (CC, such as a base station in a cellular network) owns the statistical knowledge of some spectra’s statuses, which is named the spectrum pool. As depicted by Figure 2, the spectrum pool owned by the CC has the occupied or idle status of *N* spectra in *T* time slots. This spectrum status information can be obtained by cooperative spectrum sensing through the cognitive users [21]. Meanwhile, the whole statistical status information of the spectrum pool can also be broadcasted to all cognitive users. Suppose that all users operate in a time-slotted fashion and are synchronous. To facilitate the readers, some important notations in this work are listed in Table 1.

In order to ensure a low-latency multi-hop transmission, each hop can use the idle time slot of different primary users’ frequencies. Hence, each cognitive node is supposed to be equipped with two wireless radios. In other words, each cognitive hop can transmit and receive at the same time. Simultaneously, in view of the high spectral performance, multiple hops are allowed to multiplex the same spectrum at the same time if the mutual interference is under control. Consequently, let us define $x_{k,t}^{n}\in \left\{1,0\right\}$ as whether the *k*-th hop cognitive link chooses the *n*-th spectrum to transmit or not at the *t*-th time slot. Therefore, from the above description, an intuitive constraint can be concluded:

(1) $$\sum_{k}x_{k,t}^{n}\;\leq \;x_{\max}\left(\forall t\;\in \;\left\{1,2,\;\cdots \;,T\right\},n\;\in \;\left\{1,2,\;\cdots \;,N\right\}\right).$$

Herein, $x_{\max}$ represents the maximal numbers of cognitive hops that can multiplex the same spectrum at the same time. In addition, it is assumed that each hop can only use one spectrum to transmit during its idle time. So there is another restraint for $x_{k,t}^{n}$, which can be expressed as follows:

(2) $$\sum_{n}x_{k,t}^{n}\leq 1\left(\forall t\in \left\{1,2,\cdots ,T\right\},k\in \left\{1,2,\cdots ,K\right\}\right).$$

On the basis of the above spectrum usage rules, the signal-to-interference-plus-noise-power-ratio (SINR) of the receiver node of each hop in a certain spectrum and time slot can be formulated as follows:

(3) $$SINR_{k,t}^{n}=\frac{x_{k,t}^{n}\cdot p_{k,t}^{n}\cdot g_{k,k+1}^{n,t}}{\sum_{j\neq k}x_{j,t}^{n}\cdot p_{j,t}^{n}\cdot g_{j,k+1}^{n,t}+NP_{n}},$$

where $p_{k,t}^{n}$ is the transmission power of node *k* in the *n*-th spectrum and *t*-th time slot. $NP_{n}$ is the noise power and is equal to $\left(B{}_{n}\;\cdot \;\rho_{n}\right)$. $\rho_{n}$ and $B{}_{n}$ are the noise power density and the channel bandwidth of the current spectrum, respectively. $g_{k,j}^{n,t}\left(k,j\in \left\{1,2,\cdots ,K+1\right\}\right)$ denotes the channel gain between node *k* and *j*, which accounts for the path loss and Rayleigh fading and can be expressed as:

(4) $$g_{k,j}^{n,t}=d_{k,j}^{-\lambda_{n}}\cdot \left|h_{k,j}^{n,t}\right|,$$

where $d_{k,j}$ is the physical distance between node *k* and *j*. $\lambda_{n}$ denotes the path-loss exponent of the *n*-th spectrum. $h_{k,j}^{n,t}$ is modeled as zero-mean complex Gaussian random variables with unit variance, characterizing the Rayleigh fading.

Accordingly, with the Shannon theorem, the transmission rate of each hop can be stated as

(5) $$R_{k,t}^{n}=C_{n}^{t}\cdot B_{n}\cdot \log\left(1+SINR_{k,t}^{n}\right),$$

where $C_{n}^{t}$ belongs to 0 or 1 and indicates the occupied or idle status of the PU. Obviously, from the above discussion, the set of $\left\{C_{n}^{t}\right\}$ is one part of the SSI. When the *n*-th spectrum is idle at the *t*-th time slot, it means that the *n*-th spectrum at the current time slot *t* can be used for transmission by a cognitive multi-hop link. Because any spectrum is not always occupied or idle, the spectrum idle rate can be defined to describe the availability of the *n*-th spectrum:

(6) $$v_{n}=\frac{1}{T}\sum_{t}C_{n}^{t}.$$

To better analyze the overall performance of the system, the average system utility would be considered. Consequently, (7) shows the average transmission rate per hop:

(7) $$\bar{R_{k}}=\frac{1}{T}\sum_{t}\sum_{n}R_{k,t}^{n}.$$

With the above expression of the average transmission of one hop, the average multi-hop transmission rate can be presented as follows:

(8) $$\bar{R_{SR}}=\bar{\min\left\{R_{k}\right\}},\left(\forall k\right).$$

In order to make sure that the transmission data will not be backlogged, the average transmission rate between two adjacent hops should satisfy the following constraint:

(9) $$\bar{R_{k}}\leq \bar{R_{k+1}},\left(k=\left\{1,2,\cdots ,K-1\right\}\right).$$

## 3. Mathematical Optimization Model

Based on the consideration of spectral efficiency, this work will study a spectral efficiency optimization model.

Above all, let us define $A_{n}$ as the spectrum occupancy indicator when any cognitive hop utilizes the *n*-th spectrum in any time slot. Thus, we can give a mathematical expression as demonstrated in (10) to reflect the relationship between $A_{n}$ and $x_{k,t}^{n}$.

(10) $$A_{n}=\left\{\begin{array}{c} 1,\;\mathrm{when}\;\;x_{k,t}^{n}=1\left(\forall k,t\right)\\ 0,\;x_{k,t}^{n}=0\;\mathrm{for}\;\;\mathrm{all}\;\;k\&t \end{array}\right.$$

Based on the above discussion, a spectral efficiency optimization model, which aims to investigate the optimal power and spectrum allocation strategy, in the cognitive MCMH scenario can be formulated as follows:

(11) $$\begin{array}{cc} P1: & \max_{\left\{X,P\right\}}:SE\left(X,P\right)=\max_{\left\{X,P\right\}\in \Omega }:\left\{\frac{\bar{R_{SR}}}{\sum_{n}A_{n}\cdot B_{n}}\right\}, \end{array}$$

(11a) $$\begin{array}{cc} s.t.\; & \left(1),(2),(9),(10\right) \end{array}$$

(11b) $$\begin{array}{cc} \; & \bar{R_{SR}}\geq R_{th}, \end{array}$$

(11c) $$\begin{array}{cc} \; & x_{k,t}^{n}\in \left\{0,1\right\}, \end{array}$$

(11d) $$\begin{array}{cc} \; & p_{k,t}^{n}\in \left[0,P_{k}^{\max}\right]. \end{array}$$

Herein, X,P are the sets of the spectrum occupancy indicators and the power allocation parameters, respectively. Accordingly, (11b) denotes the transmission rate requirement constraint of the multi-hop link. $R_{th}$ is the minimal transmission rate threshold. (11c) and (11d) represent the value range constraints of $x_{k,t}^{n}$ and $p_{k,t}^{n}$.

From the description of the problem **P1**, we can clearly see that the optimization variables include some real-valued (P) and binary-valued (X) parameters. In addition, with the combination of the non-convex transmission rate expression (5) and the indirect spectrum occupancy indicator $A_{n}$, the modeled optimization problem is a complex non-convex fractional mixed integer nonlinear programming (MINLP) problem. This type of issue can easily prove to be NP-hard from the computational complexity of MILP [22]. Despite the complexity, this work proposes a successive multi-step convex approximation scheme (SMCA) to solve the modeled problem.

## 4. The Proposed Successive Multi-Step Convex Approximation Scheme

The SMCA scheme contains three main steps. First of all, we utilize the Dinkelbach algorithm [23] to equivalently transform the fractional objective utility formula (11) to a multi-objective program. Then, the non-convex constraints and parameters are relaxed into the corresponding convex expressions. Finally, a valid and convex matrix norm is defined to describe the indirect relationship between $A_{n}$ and $x_{k,t}^{n}$. As a result, the modeled non-convex fractional MINLP problem **P1** can be converted into a convex one and solved by a typical convex optimization method, such as the Newton algorithm. The details of these three main steps will be described in the next sections.

### 4.1. The Fractional Equivalent Conversion Method

The target of this section is transforming the fractional objective utility formula (11) into a multi-objective program, which is easier to solve. For convenience, let us denote Ω as the feasible solution set of the problem **P1** and $q^{*}$ as the optimal value of spectral efficiency. Notably, we can get the reasonable expression of $q^{*}$ as follows:

(12) $$\begin{array}{c} q^{*}=\max_{\left\{X,P\right\}\in \Omega }:SE\left(X,P\right)=\max_{\left\{X,P\right\}\in \Omega }:\left\{\frac{\bar{R_{SR}}}{\sum_{n}A_{n}\cdot B_{n}}\right\}\\ \;=SE\left(X^{*},P^{*}\right)={\left\{\frac{\bar{R_{SR}}}{\sum_{n}A_{n}\cdot B_{n}}\right\}}_{\left\{X^{*},P^{*}\right\}}, \end{array}$$

where $X^{*}$ and $P^{*}$ are the optimal solutions of problem **P1**. Subsequently, the following Theorem can be presented according to the Dinkelbach algorithm [23]:

**Theorem** **1.** The optimal spectral efficiency $q^{*}$ can be achieved if and only if:

(13) $$\max_{\left\{X,P\right\}\in \Omega }:\left\{\bar{R_{SR}}-q^{*}\sum_{n}A_{n}\cdot B_{n}\right\}\to 0\Leftrightarrow {\left\{\bar{R_{SR}}-q^{*}\sum_{n}A_{n}\cdot B_{n}\right\}}_{\left\{X^{*},P^{*}\right\}}=0,$$

*where $\forall \left\{X,P\right\}\in \Omega$ makes ${\left\{\bar{R_{SR}}\right\}}_{\left\{X,P\right\}}\geq 0$ and ${\left\{\sum_{n}A_{n}\cdot B_{n}\right\}}_{\left\{X,P\right\}}>0$.*

**Proof:**  The above Theorem is proved in Appendix A in a similar way as [23]. □

Hence, (13) can be handled by an iterative process, which is demonstrated by Algorithm 1. Define *m* as the number of iterations, $q^{m}$ as the instantaneous SE in the *m*-th iteration, and ε as the convergence threshold. 

**Algorithm 1:** out-layer iterative algorithm**Initialization:**m=1, $q^{m}=0$, $\varepsilon =10^{-2}$; step 1: For a given $q^{m}$, obtain $X^{\prime }$ and $P^{\prime }$ by solving the following optimization problem:
(14)$$\begin{array}{c} F\left(q^{m}\right)=\max_{\left\{X,P\right\}\in \Omega }:\left\{\bar{R_{SR}}-q^{m}\sum_{n}A_{n}\cdot B_{n}\right\}. \end{array}$$ step 2: Update $q^{m}=SE\left(X^{\prime },P^{\prime }\right)=\frac{\bar{R_{SR}}}{\sum_{n}A_{n}\cdot B_{n}}$;m=m+1. step 3: $\left\{\begin{array}{c} Execute\;steps\;1∼2,if\left|F\left(q^{m}\right)\right|>\varepsilon \\ Return\;\left(X^{\prime },P^{\prime }\;\right),f\left|F\left(q^{m}\right)\right|\leq \varepsilon . \end{array}\right.$


### 4.2. The Effective Convex Relaxation Scheme

The design of the convex relaxation scheme aims to solve the non-convex equations and variables of problem **P1**. Hence, there are four primary solutions to handle the non-convex equations and variables.

#### 4.2.1. Continuous Discrete Variables X

It is hard to directly solve the set of binary variables X. So, we relax the range of X from 0 to 1, i.e., $X=\left\{x_{k,t}^{n}\right\}\in \left[0,1\right]$.

#### 4.2.2. The Convexification of Transmission Rate Expression

From the spectrum usage rules and the expression of the transmission rate of the multi-hop (5), it is easy to find out that the set of transmission rates $R=\left\{R_{k,t}^{n}\right\}$ is not convex according to the second-order criterion of convex functions. Nevertheless, a three-step convex relaxation scheme can transform the non-convex transmission rate into a convex one.

- The first step replaces $x_{k,t}^{n}\cdot p_{k,t}^{n}$ with the equivalent $S_{k,t}^{n}$. The physical meaning of this substitution has two aspects, which can be intuitively expressed as the following two equations:
  (15) $$S_{k,t}^{n}=x_{k,t}^{n}\cdot p_{k,t}^{n},$$
  (16) $$x_{k,t}^{n}=\left\{\begin{array}{c} 1,\;\mathrm{if}\;S_{k,t}^{n}>0\;\\ 0,\;\mathrm{if}\;S_{k,t}^{n}\leq 0\; \end{array}\right..$$
- The second step introduces a convex approximation formula shown as inequality (17) to acquire the approximate transmission rate of the original one.
  (17) αlogn+β≤log(1+n)
  The above approximation is proven to be tight and has low complexity when $n\;=\;n_{0}$ and $\alpha \;=\;\frac{n_{0}}{1\;+\;n_{0}},\beta \;=\;\log\left(1\;+\;n_{0}\right)\;-\;\alpha \log n_{0}$ [24].
- The third step involves performing some equivalent substitution of variables $S_{k,t}^{n}$ by equations of $S_{k,t}^{n}=e^{\tilde{S_{k,t}^{n}}}$.

After applying the above three steps to the original transmission rate $R=\left\{R_{k,t}^{n}\right\}$, we can obtain the corresponding approximation expression, as (18) reveals.

(18) $$\tilde{R_{k,t}^{n}}\left(\tilde{S_{k,t}^{n}}\right)=C_{n}^{t}\cdot B_{n}\cdot \left\{\alpha_{k,t}^{n}\left[\tilde{S_{k,t}^{n}}+\log\left(g_{k,k+1}^{n,t}\right)-\log\left(\sum_{j\neq k}e^{\tilde{S_{j,t}^{n}}}\cdot g_{j,k+1}^{n,t}+NP_{n}\right)\right]+\beta_{k,t}^{n}\right\}$$

From (18), the update rules of $\alpha_{k,t}^{n},\beta_{k,t}^{n}$ are the same as [24]. Obviously, according to the convexity of *log-sum-exp* [25], (18) can easily prove to be a concave one with parameter $\tilde{S_{k,t}^{n}}$. Apparently, after replacing $\tilde{R_{k,t}^{n}}$ with $R_{k,t}^{n}$, the single-hop average transmission rate as shown in formula (7), which is the sum of concave functions, is also concave. Meanwhile, it is easy to prove that the minimum value of multiple concave functions is also concave (see Appendix B).

#### 4.2.3. The Relaxation of Non-Convex Constraints

The discontinuity and non-convexity can be solved by Section 4.2.1 and Section 4.2.2, though some constraints, such as (9) and (16), also have non-convex properties. Based on this, we will perform the relevant convexification operations one by one.

**Figure 3:**
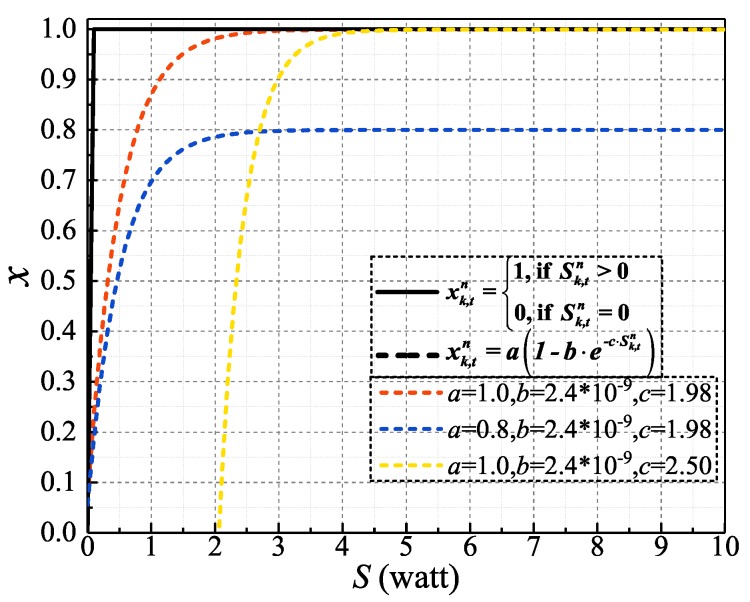
Concave approximation for the Heaviside step function.

- For (9):
  As described in Section 4.2.2, the single-hop average transmission rate is concave. (9) is clearly a difference of convex functions (DC). In response to the DC constraint, let us simply turn the inequality constraint into an equation: $\bar{R_{k}}=\bar{R_{k+1}},\left(k=\left\{1,2,\cdots ,K-1\right\}\right)$. With this, it is easy to solve this equation case.
  First of all, let us define a variable $\overset{\Delta }{R}$ to denote the final value of $\bar{R_{k}}$:
  (19) $$\overset{\Delta }{R}=\bar{R_{k}}\left(k=\left\{1,2,\cdots ,K\right\}\right).$$
  Then, the expression (19) is equivalent to solving the following problem:
  (20) $$\begin{array}{cc} P2: & \min_{\overset{\Delta }{R},\gamma }:\sum_{k}\left(\gamma_{k}\right), \end{array}$$
  (20a) $$\begin{array}{cc} s.t.\; & \bar{R_{k}}-\overset{\Delta }{R}\geq \gamma_{k}\left(k=\left\{1,2,\cdots ,K\right\}\right), \end{array}$$
  (20b) $$\begin{array}{cc} \; & \overset{\Delta }{R},\gamma =\left\{\gamma_{k}\right\}\geq 0. \end{array}$$
  From problem **P2**, we can see that it is not only convex programming but can also get $\overset{\Delta }{R}$ and $\bar{R}=\left\{\bar{R_{k}}\right\}$ as close as possible.
- For (16):
  Equation (16) shows the relationship between the spectrum usage indicator $x_{k,t}^{n}$ and the power allocation parameter $S_{k,t}^{n}$. It is quite clear that (16) is a Heaviside step function, which is neither convex nor concave. However, the value range of $S_{k,t}^{n}$ is no less than 0. This means that the relationship between $x_{k,t}^{n}$ and $S_{k,t}^{n}$ can be depicted by the black line in Figure 3. With this, a mathematical expression demonstrated by (21) can intuitively describe the relationship between $x_{k,t}^{n}$ and $S_{k,t}^{n}$:
  (21) $$x_{k,t}^{n}=a\left(1-b\cdot e^{-c\cdot S_{k,t}^{n}}\right),$$
  where *a*, *b*, *c* are constants and are greater than or equal to zero.
  The dotted lines in Figure 3 display the difference manifestations of (21) under different parameter ($\left\{a,b,c\right\}$) settings. Apparently, *a* controls the scaling of the *Y*-axis value, and b,c can make the controls the displacement of the *X*-axis values.
  With regard to the convex equality constraint, we hope that the final result of the both sides of (21) should be kept as close as possible. With this, problem **P2** gives the corresponding solution. In a similar way, let $d_{k,t}^{n}$ represent the distance between the expressions on both sides of (21). Thus, we can obtain a feasible convex approximate expression, as the problem **P3** shows for (21).
  (22) $$\begin{array}{cc} P3: & \min_{D}:\sum_{n}\sum_{t}\sum_{k}\left(d_{k,t}^{n}\right), \end{array}$$
  (22a) $$\begin{array}{cc} s.t.\; & a\left(1-b\cdot e^{-c\cdot S_{k,t}^{n}}\right)-x_{k,t}^{n}\geq d_{k,t}^{n}, \end{array}$$
  (22b) $$\begin{array}{cc} \; & D=\left\{d_{k,t}^{n}\right\}\geq 0. \end{array}$$

### 4.3. The Custom Convex Matrix Norm

The relationship between $A_{n}$ and $x_{k,t}^{n}$ as depicted by (10) cannot be directly applied to the modeled spectral efficiency problem. Aiming at resolving this issue, a custom convex matrix norm, which is defined as expression (23), is proposed to intuitively describe the relationship between $A_{n}$ and $x_{k,t}^{n}$.

(23) $${∥A∥}_{n}=\max_{\forall k,t}\left(\left|x_{k,t}^{n}\right|\right)$$

**Theorem** **2.** *The given norm (23) is a standard matrix norm and is convex.*

**Proof:**  The proof of this theorem is provided in Appendix C. □

### 4.4. The Final Expression of Problem ***P1***

By integrating the above convex approximation operations of Section 4.1, Section 4.2 and Section 4.3, the original average SE problem **P1** can be transformed as follows:

(24) $$\begin{array}{cc} P: & \max_{X,\tilde{S},\overset{\Delta }{R},\gamma ,D}\;:\;\tilde{F}\left(q^{m}\right)\;-\;\sum_{k}\left(\gamma_{k}\right)\;-\;\sum_{n}\sum_{t}\sum_{k}\left(d_{k,t}^{n}\right), \end{array}$$

(24a) $$\begin{array}{cc} s.t.\; & \bar{R_{SR}^{∼}}=\min\left\{\bar{R_{k}^{∼}}\right\}=\min\left\{\frac{1}{T}\sum_{t}\sum_{n}\tilde{R_{k,t}^{n}}\right\}, \end{array}$$

(24b) $$\begin{array}{cc} \; & \sum_{k}x_{k,t}^{n}\leq x_{\max}\left(\forall t,n\right), \end{array}$$

(24c) $$\begin{array}{cc} \; & \sum_{n}x_{k,t}^{n}\leq 1\left(\forall t,k\right), \end{array}$$

(24d) $$\begin{array}{cc} \; & \bar{R_{k}^{∼}}-\overset{\Delta }{R}\geq \gamma_{k}\left(\forall k\right), \end{array}$$

(24e) $$\begin{array}{cc} \; & a\left(1-b\cdot e^{-c\cdot e^{\tilde{S_{k,t}^{n}}}}\right)-x_{k,t}^{n}\geq d_{k,t}^{n}\left(\forall k,t,n\right), \end{array}$$

(24f) $$\begin{array}{cc} \; & X=x_{k,t}^{n}\in \left[0,1\right], \end{array}$$

(24g) $$\begin{array}{cc} \; & \tilde{S}=\tilde{S_{k,t}^{n}}\leq \log\left(P_{k}^{\max}\right), \end{array}$$

(24h) $$\begin{array}{cc} \; & \overset{\Delta }{R},\gamma =\gamma_{k},D=d_{k,t}^{n}\geq 0. \end{array}$$

Herein, $\tilde{F}\left(q^{m}\right)=\bar{R_{SR}^{∼}}-q^{m}\sum_{n}{∥A∥}_{n}\;\cdot \;B_{n}$. Clearly, problem **P** is a convex programming, which can solved by the typical convex optimization algorithms. By combining with the Dinkelbach’s fractional programming scheme shown as Algorithm 1, we can obtain the sub-optimal solutions for problem **P1**.

## 5. Simulation Results

The goals of this section are to verify the effectiveness of our proposed scheme and to study the network performance, including the average SE, in different scenarios.

Consequently, we will first of all present numerical results to evaluate the proposed scheme—SMCA—in aspects of average (avg.) achievable SE, transmission rate of multi-hop, number of spectrum occupation, and execution time, by comparing with three common algorithms: the random access strategy (RAS), the exhaustive searching scheme (ESS), and a heuristic algorithm: genetic algorithm (GA).

In RAS [15], once each hop detects the idle spectrum, it will access the spectrum to transmit with a certain probability. If multiple hops choose to access the same idle spectrum at the same time, the access strategy will be invalid when the transmission rate of each hop cannot be satisfied. For ESS, it will enumerate all possible access solutions and finally find the optimal solution that can maximize the average SE problem **P1**. In view of GA, we code the solutions $\left\{X,P\right\}$ as the gene sequences of each generation by the binary and real way, respectively. Meanwhile, the original SE objective of problem **P1** will be modeled as the utility function. GA can obtain a sub-optimal solution of problem **P1** by crossing, mutating, and iterating the gene sequences.

Subsequently, the performance of an MRMH CRN is analyzed along with the change of the spectrum idle rate. Based on the analysis results, we can obtain some potential deployment rules for MRMH CRNs under multi-slot scenarios in the near future.

### 5.1. Simulation Set-Up

Above all, the performance of the compared methods and the proposed algorithms in this work are evaluated via Matlab simulations. The involved convex optimization is cvx-mosek toolbox. As illustrated by Figure 2, the considered cognitive multi-hop multi-channel transmission scenario is a square that has a side length of 1000 m. The CC has the capability of acquiring all users’ positions, statistical channel status information (CSI), and spectrum status information (SSI). All nodes of the multi-hop link can randomly distribute in the square. In the multi-hop link, each node can only communicate with the nearest node. So, suppose that the distance between the transmitter and receiver of each hop is randomly selected between [200, 250] m. The spectral idle rate of each spectrum is assumed to be an i.i.d sample. The other network parameters used in this work are listed in Table 2.

We repeat each simulation scenario with the same parameter settings (for example *K* is 2) 100 times and average the results. In addition, there are some parameter settings of GA should be illustrated. According to some typical research works on GAs [28], the probabilities of crossover and mutation are 0.8 and 0.02, respectively. The population size of each generation and the number of populations are set as 30 and 1000, respectively.

### 5.2. Complexity Comparison

As we all know, thoroughly and correctly analyzing a complex convex nonlinear programming problem is difficult. However, generally speaking, the computational complexity is related to the running time of an algorithm until a solution is found [29]. Moreover, there are some commonalities among the above-mentioned algorithms.

According to the principles of the algorithms (SMCA, RAS, ESS, GA) and the simulation environment, we can see that the proposed algorithm (SMCA) is an ε-iteration convex optimization scheme, and the convex optimization contains the spectrum and power allocation. For RAS and ESS, both of them firstly confirm the spectrum allocation and then implement the power allocation. The difference between RAS and ESS is that RAS explores feasible resource allocation and ESS is committed to investigating optimal resource allocation. Furthermore, the computational complexity of the typical GA is positively correlated with the product of the number of objectives ($N_{O}$) and the population size ($P_{S}$) [30]. Simultaneously, assume that the computational complexities of the spectrum and power allocation are $O\left(S\right)$ and $O\left(P\right)$, respectively. Table 3 summarizes the complexities of all involved algorithms.

In Table 3, *V* denotes the overall average spectrum idle rate and is equal to $\frac{1}{N}\sum_{n}v_{n}$. From Table 3, we can see that the computational complexity of ESS will grow exponentially with the increase in the number of users, spectra, and time slots. Therefore, ESS has the highest complexity. In addition, due to the fact that RAS can explore a feasible solution that satisfies all constraints, RAS is the lightest algorithm. Moreover, SMCA transforms the non-convex fractional MINLP problem into a feasible convex successive NLP problem, which includes the spectrum and power allocation at the same time. Meanwhile, the transformed problem can be solved by the ε-iteration convex optimization algorithm. Consequently, SMCA exhibits a little bit higher complexity than RAS and is much lower than ESS. In terms of GA, $N_{O}$ denotes the number of objectives, which includes the resource allocation strategies (*X* and *P*). By combining with the population size $P_{S}$ and the gene iteration procedure, the complexity of GA will get close to ESS. Thus, the order of complexity of the involved algorithms, from high to low, is: ESS, GA, SMCA, and RAS.

After analyzing the computational complexity of the involved algorithms, the next sections will discuss the algorithms’ performance under different scenarios with different spectral idle rates. Thus, initially, considering the complexity of ESS, we will investigate the effectiveness of the proposed algorithm—SMCA—by comparing it with ESS and RAS in single-hop scenario. What’s more, the performance of the algorithms (SMCA and RAS) is not only well-verified in multi-hop scenarios, but the relationship between network performance and spectrum idle rate can also be well-explored, which can give us some bright deployment strategies for the multi-hop, -channel, and -slot scenarios.

### 5.3. Performance Results and Analysis

#### 5.3.1. One-Hop Simulation Scenario

**Figure 4:**
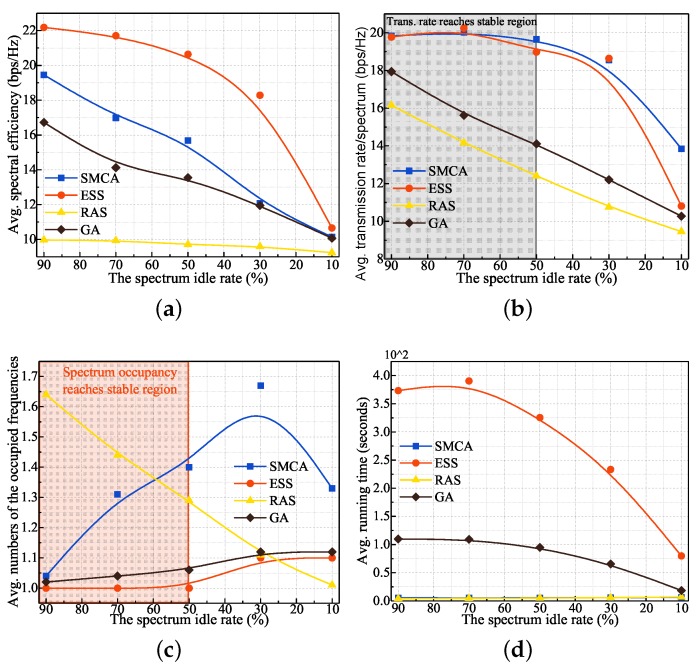
Performance results of the involved algorithms under different spectrum idle rates in a one-hop scenario. (**a**) spectrum efficiency (**b**) one-hop transmission rate (**c**) number of spectra occupied (**d**) execution time.

The performance results in terms of average spectrum efficiency, multi-hop transmission rate, spectrum occupation, and execution time of the involved algorithms as the spectrum idle rate changes are vividly demonstrated by Figure 4. Notably, the execution time of GA is the time when the stable SE is obtained. As we can see from Figure 4, there are three valid phenomena that can be observed:

- As demonstrated by Figure 4, the trend for most performances will gradually decline as the spectrum idle rate decreases. Above all, the downtrend of the transmission rate is obvious because the available spectrum resources reduce with the decrease in the spectrum idle rate. To achieve the maximum SE, the number of occupied frequencies attempts to keep stable. As a result, the average SE will go down. For execution time, the feasible solution space gets bigger when the spectrum idle rate increases. At this moment, the time to find the optimal solutions goes up accordingly.
- From the results of Figure 4a–c, the performance of SMCA can get close to the optimal performance of ESS, especially when the spectrum idle rate reaches 50% or more. The performance difference between SMCA and ESS is mainly because SMCA is a lower-bounded convex approximation scheme of the original problem. Although GA has the closest spectrum occupancy performance to ESS, GA has a poor performance compared to SMCA in terms of transmission rate and spectrum efficiency. That is because the exploration space of the binary value (X) is lower or smaller than the real value (P). Thus, the performance of the average transmission rate and SE, which is related to both X and P, is relatively inadequate for obtaining the best result. This phenomenon can be explained visually with the performance results when the spectrum idle rates are 10% and 90%. As depicted by Figure 4a, the performance difference in SE when the spectrum idle rate equals 10% is relatively smaller than 90%. This is due to the fact that the feasible solution space needed to explore is smaller under the spectrum idle rate of 10%. At this moment, GA can obtain a better result faster and easier. When the feasible solution space increases under the spectrum idle rate of 90%, GA needs more time to find the optimal solution and may be more likely to fall into a local optimal solution. However, looking at the execution times of the algorithms, ESS takes a long time to find the optimal solution and GA follows ESS closely. By comparing the running times of SMCA and RAS, the theoretical and practical computational complexity of ESS is the main obstacle in network optimization. In addition, the performance of ESS is unsatisfactory, though ESS has the shortest execution speed. The performance variances in terms of average SE and transmission rate between RAS and SMCA can reach about 10–50%. Therefore, SMCA can not only obtain a sub-optimal solution of the optimal one for the modeled SE problem but also has a lower computational complexity.
- From the performance results of ESS and SMCA, some potential relationships between network performance and spectrum idle rate can be found. The overall performance trend will tend to be stable when the spectrum idle rate is no less than 50%. This phenomenon is particularly evident in the average number of spectra occupied and transmitting rate performance. Meanwhile, the increasing rate of the average SE is just 7% when the spectrum idle rate grows from 50% to 90%. Consequently, we can come to the conclusion that the stable performance of multi-channel, -radio, and -slot CRN can be obtained when the spectrum idle rate reaches 50% or more in signal-node scenarios.

#### 5.3.2. Multi-Hop Simulation Scenario

**Figure 5:**
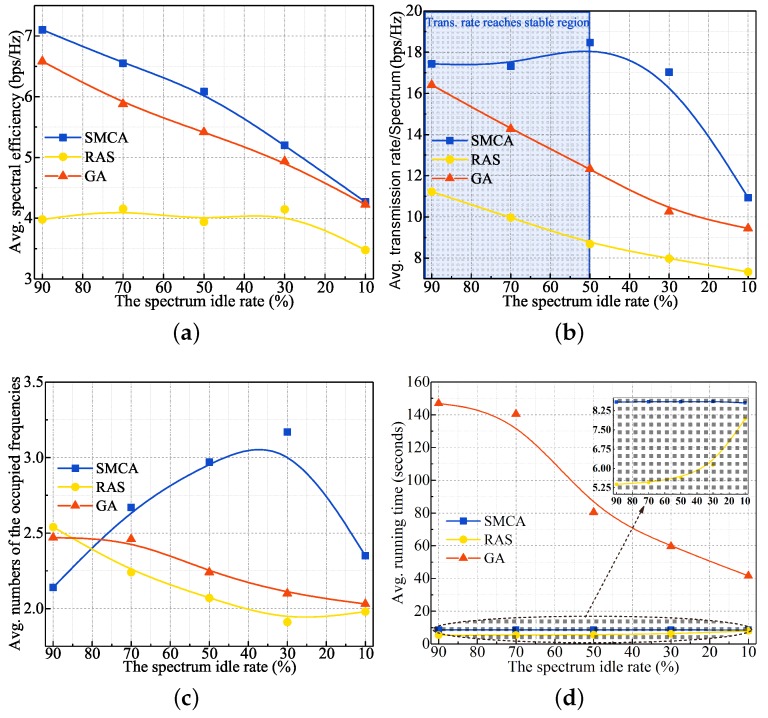
Performance results of the involved algorithms under different spectrum idle rates multi-hop scenario. (**a**) spectrum efficiency (**b**) multi-hop transmission rate (**c**) number of spectra occupied (**d**) execution time.

Considering the higher complexity of ESS, we only study the performance in multi-hop simulation scenarios to verify the effectiveness of the involved algorithms: SMCA, RAS, and GA. Just like the performance results in one-hop, GA and RAS have better spectrum occupancy performance than SMCA. But SMCA has the best transmission rate and spectrum efficiency performance. As depicted by Figure 5, the performance variances in terms of average SE and transmission rate between SMCA and RAS can reach about 20–50%. The main reason for this phenomenon is that RAS only explores a feasible solution that can satisfy all constraints. Moreover, the performance difference between SMCA and GA can be up to 11–40% in the case of average SE and transmission rate, respectively.

However, the maximum average execution time of SMCA and RAS is less than 10 s, and GA can reach more than 100 s. In addition, the difference in average execution time is only 3 s between SMCA and RAS in the worst case. So we can draw a valid conclusion that SMCA can be used as a feasible optimization scheme for multi-hop multi-channel scenarios due to its good performance and a tolerable computational overhead.

## 6. Conclusions

This work addressed the spectrum and power allocation simultaneously by modeling an average SE optimization problem for a cognitive multi-hop link. Despite the complex non-convex fractional MINLP characteristic, this work proposed an SMCA to transform the modeled problem into a successive convex one. From the theoretical computational complexity and simulation analysis, the proposed SMCA could obtain a sub-optimal network performance by comparing with ESS and had a lower complexity that was very close to RAS. As a result, the introduced SMCA could be an effective solution to apply to study the complex non-convex MINLP problem of MRMH CRNs. Furthermore, the stable area of network performance was investigated along with the increasing of spectrum idle rate, which provided us with some bright references about the design of MRMH CRNs.

## Figures and Tables

**Table 1:** Important notations.

Symbol	Definition
*K*	Total number of cognitive hops
*T*	Total number of time slots
*N*	Total number of spectra
*k*	*k*-th cognitive hop, which belongs to [1,K]
*t*	*t*-th time slot, where $t\in \left[1,T\right]$
*n*	*n*-th spectrum, where $n\in \left[1,N\right]$
$x_{k,t}^{n}$	Whether the *k*-th hop chooses to use the *n*-th spectrum in the *t*-th time slot
$x_{max}$	Maximum number of hops that can use the same spectrum to transmit at the same time
$SINR_{k,t}^{n}$	Signal to interference plus noise power when the *k*-th hop chooses to use the *n*-th spectrum in the *t*-th time slot
$p_{k,t}^{n}$	Transmission power of the *k*-th hop when it chooses to use the *n*-th spectrum in the *t*-th time slot
$P_{k}^{max}$	Maximum transmission power of each hop in each time slot
$g_{k,j}^{n,t}$	Channel gain between node *k* and *j*, where $k,j\in \left[1,K+1\right]$
$d_{k,j}$	Physical distance between node *k* and *j*
$\lambda_{n}$	Path-loss exponent of the *n*-th spectrum
$h_{k,j}^{n,t}$	Rayleigh fading, which obeys a Gaussian distribution
$NP_{n}$	Noise power
$B_{n}$	Channel bandwidth of each spectrum
$\rho_{n}$	Density of noise power of the *n*-th spectrum
$R_{k,t}^{n}$	Transmission rate of the *k*-th hop under the *n*-th spectrum and the *t*-th time slot
$C_{n}^{t}$	Whether the spectrum is idle or occupied by primary users
$v_{n}$	Spectrum idle rate under *T* time slots
$\bar{R_{k}}$	Average transmission rate of the *k*-th hop
$\bar{R_{SD}}$	Average transmission rate of the multi-hop link
$A_{n}$	Whether the *n*-th spectrum is idle or occupied by a cognitive link
$R_{th}$	Minimum transmission rate threshold of the multi-hop link

**Table 2:** Parameter settings in the multi-channel multi-hop (MCMH) simulation environment [26,27].

Patameter	Value
Path loss of each hop ($\lambda_{n}$)	[3, 5]
Max Tx power of each hop ($P_{k}^{max}$)	40 dBm
Spectrum idle rate ($v_{n}$)	[0.1, 0.9]
Channel bandwidth ($B_{n}$)	[100, 150] KHz
Noise power density ($\rho_{n}$)	−174 dBm/Hz
Number of hops (*K*)	[1, 2]
Number of spectra (*N*)	10
Number of time slots (*T*)	2
Transmission rate threshold ($R_{th}$)	2 bps/Hz
Maximum number of hops that can multiplex the same spectrum ($x_{max}$)	3

**Table 3:** The computational complexity of successive multi-step convex approximation (SMCA), the random access strategy (RAS), and the exhaustive searching scheme (ESS).

Algorithm	Complexity
SMCA	$O\left(S\cdot P\cdot \log\left(\frac{1}{\varepsilon }\right)\right)$
RAS	$O\left(P\right)$
ESS	$O\left(2^{\min\left(x_{\max},N\right)\cdot V\cdot K\cdot T}\cdot P\right)$
GA	$O\left(N_{O}\cdot P_{S}\right)$

## Appendix A. Proof of Theorem 1

First of all, the forward implication of Theorem 1 should be proved. As defined in Section 4.1, $q^{*}$ and $\left\{X^{*},P^{*}\right\}\in \Omega$ are the optimal SE and resource allocation solutions of the problem **P1**, respectively. Obviously, the following expression of the optimal SE can be obtained:

(A1) $$q^{*}={\left\{\frac{\bar{R_{SR}}}{\sum_{n}A_{n}\cdot B_{n}}\right\}}_{\left\{X^{*},P^{*}\right\}\in \Omega }\geq {\left\{\frac{\bar{R_{SR}}}{\sum_{n}A_{n}\cdot B_{n}}\right\}}_{\left\{X,P\right\}\in \Omega }.$$

Then, we have the following expressions according to formula (A1):

(A2) $$\left\{\begin{array}{c} {\left\{\bar{R_{SR}}-q^{*}\sum_{n}A_{n}\cdot B_{n}\right\}}_{\left\{X,P\right\}\in \Omega }\leq 0,\\ {\left\{\bar{R_{SR}}-q^{*}\sum_{n}A_{n}\cdot B_{n}\right\}}_{\left\{X^{*},P^{*}\right\}\in \Omega }=0. \end{array}\right.$$

It is quite clear that the optimal resource allocation solutions $\left\{X^{*},P^{*}\right\}$ can be achieved by maximizing the target function: ${\left\{\bar{R_{SR}}-q^{*}\sum_{n}A_{n}\cdot B_{n}\right\}}_{\left\{X,P\right\}\in \Omega }$ and making the target function tend to 0. Consequently, this completes the forward implication.

Secondly, we prove the converse implication of Theorem 1. Suppose that $\left\{X^{*},P^{*}\right\}\in \Omega$ are the optimal resource allocation solutions of the problem **P1**. When $q^{*}$ is the optimal SE, we can obtain

(A3) $${\left\{\bar{R_{SR}}-q^{*}\sum_{n}A_{n}\cdot B_{n}\right\}}_{\left\{X^{*},P^{*}\right\}\in \Omega }=0.$$

Therefore, for any $\left\{X,P\right\}\in \Omega$, we have the following inequality:

(A4) $${\left\{\bar{R_{SR}}-q^{*}\sum_{n}A_{n}\cdot B_{n}\right\}}_{\left\{X,P\right\}\in \Omega }\leq {\left\{\bar{R_{SR}}-q^{*}\sum_{n}A_{n}\cdot B_{n}\right\}}_{\left\{X^{*},P^{*}\right\}\in \Omega }.$$

The preceding inequality implies that $q^{*}={\left\{\frac{\bar{R_{SR}}}{\sum_{n}A_{n}\cdot B_{n}}\right\}}_{\left\{X^{*},P^{*}\right\}\in \Omega }\geq {\left\{\frac{\bar{R_{SR}}}{\sum_{n}A_{n}\cdot B_{n}}\right\}}_{\left\{X,P\right\}\in \Omega }$.

In other words, the optimal solutions $\left\{X^{*},P^{*}\right\}\in \Omega$ of the Equation (A3) are also the optimal resource allocation policies of problem **P1**.

## Appendix B. Concave Proof of the Minimum Value of Two Concave Functions

Let us define the minimum value of two concave functions $f\left(x\right)$ and $g\left(x\right)$ as $L\left(x\right)$, i.e., $L\left(x\right)=\min\left\{f\left(x\right),g\left(x\right)\right\}$, where x∈ℜ.

According to the concave definition, for concave functions $f\left(x\right)$ and $g\left(x\right)$, we have:

$$\left\{\begin{array}{c} a\cdot f\left(x\right)+\left(1-a\right)\cdot f\left(y\right)\leq f\left(z\right),\\ a\cdot g\left(x\right)+\left(1-a\right)\cdot g\left(y\right)\leq g\left(z\right), \end{array}\right.$$

where $z=a\cdot x+\left(1-a\right)\cdot y$ and the range of *a* is between 0 and 1.

Therefore, we can derive the following inequality:

$$\begin{array}{c} a\min\left\{f\left(x\right),g\left(x\right)\right\}+\left(1-a\right)\min\left\{f\left(y\right),g\left(y\right)\right\}\leq a\cdot f\left(x\right)+\left(1-a\right)\cdot f\left(y\right)\leq f\left(z\right). \end{array}$$

Similarly,

$$\begin{array}{c} a\min\left\{f\left(x\right),g\left(x\right)\right\}+\left(1-a\right)\min\left\{f\left(y\right),g\left(y\right)\right\}\leq a\cdot g\left(x\right)+\left(1-a\right)\cdot g\left(y\right)\leq g\left(z\right). \end{array}$$

Thus, we can obtain the following inequality:

$$\begin{array}{c} a\min\left\{f\left(x\right),g\left(x\right)\right\}+\left(1-a\right)\min\left\{f\left(y\right),g\left(y\right)\right\}\leq \min\left\{f\left(z\right),g\left(z\right)\right\} \end{array}$$

As discussed above, we can find out that the minimum value of two concave functions is concave on the basis of the definition of a concave function.

## Appendix C. Convex Proof of the Custom Matrix Norm

Before handling the convexity of the custom norm (23), we should prove that the given norm is a standard matrix norm. Thus, the proof can be done by discussing the four features of a standard matrix norm:

- Non-negativity:
  $$\begin{array}{c} \mathrm{when}:\;\exists n,\forall k,t\Rightarrow x_{k,t}^{n}\geq 0,\\ \mathrm{so}:\;\;\;\;\;\exists k^{*},t^{*}\Rightarrow {∥A∥}_{n}=\max_{\forall k,t}\left(\left|x_{k,t}^{n}\right|\right)=x_{k^{*},t^{*}}^{n}\geq 0. \end{array}$$
  From the above demonstration, we can conclude that the custom norm (23) is non-negative.
- Zero matrix:
  Firstly:
  $$\begin{array}{c} \mathrm{when}:\;\exists n,\forall k,t\Rightarrow x_{k,t}^{n}=0,\\ \mathrm{so}:\;\;\;\;\;{∥A∥}_{n}=\max_{\forall k,t}\left(\left|x_{k,t}^{n}\right|\right)=0. \end{array}$$
  Secondly:
  $$\begin{array}{c} \mathrm{when}:\exists n,\forall k,t\;\Rightarrow \;{∥A∥}_{n}\;=\;\max_{\forall k,t}\left(\left|x_{k,t}^{n}\right|\right)\;=\;0\&x_{k,t}^{n}\in \left[0,1\right],\\ \mathrm{so}:\;\;\;\;\;\exists n,\forall k,t\Rightarrow x_{k,t}^{n}=0. \end{array}$$
  Consequently, the custom norm (23) is a zero matrix when $x_{k,t}^{n}$ is equal to zero.
- Homogeneity:
  $$\begin{array}{c} \mathrm{when}:\exists a\in ℜ,\exists n,\forall k,t\Rightarrow {∥A∥}_{n}=\max_{\forall k,t}\left(\left|x_{k,t}^{n}\right|\right),\\ \mathrm{so}:\;\;\;\;\;{∥a\cdot A∥}_{n}=\max_{\forall k,t}\left(\left|a\cdot x_{k,t}^{n}\right|\right)=\left|a\right|\cdot \max_{\forall k,t}\left(\left|x_{k,t}^{n}\right|\right)=\left|a\right|\cdot {∥A∥}_{n}. \end{array}$$
  Thus, the custom norm (23) satisfies homogeneity.
- Triangle inequalities:
  $$\begin{array}{c} \mathrm{assume}:\exists n,\forall k,t\Rightarrow x_{k,t}^{n},y_{k,t}^{n}\in \left[0,1\right],\mathrm{and}\;\mathrm{define}:{∥A∥}_{n}=\max_{\forall k,t}\left(\left|x_{k,t}^{n}\right|\right),{∥B∥}_{n}=\max_{\forall k,t}\left(\left|y_{k,t}^{n}\right|\right),\\ \mathrm{so}:\;{∥A+B∥}_{n}=\max_{\forall k,t}\left(\left|x_{k,t}^{n}+y_{k,t}^{n}\right|\right)\leq \max_{\forall k,t}\left(\left|x_{k,t}^{n}\right|\right)+\max_{\forall k,t}\left(\left|y_{k,t}^{n}\right|\right)={∥A∥}_{n}+{∥B∥}_{n}. \end{array}$$

As discussed above, we can clearly see that the custom norm meets the four typical features of a matrix norm. In other words, the self-defined norm is a matrix norm.

After that, we should prove the convexity of the matrix norm.

Firstly, let us define a function $f\left(x\right)=\max_{\forall k,t}\left(\left|x_{k,t}^{}\right|\right)$. Assume that there exists a constant *a*. From this, we can obtain the expression of $f\left(a\cdot x+\left(1-a\right)\cdot y\right)$ as follows:

$$\begin{array}{c} f\left(a\cdot x+\left(1-a\right)\cdot y\right)=\max_{\forall k,t}\left(\left|c_{k,t}^{}\right|\right),\left(c_{k,t}^{}=a\cdot x_{k,t}^{}+\left(1-a\right)\cdot y_{k,t}^{}\right). \end{array}$$

According to the triangle inequalities and the homogeneity of the custom matrix norm, the above formula can be indicated as

$$\begin{array}{c} f\left(a\cdot x+\left(1-a\right)\cdot y\right)=\max_{\forall k,t}\left(\left|c_{k,t}^{}\right|\right)=\max_{\forall k,t}\left(\left|a\cdot x_{k,t}^{}+\left(1-a\right)\cdot y_{k,t}^{}\right|\right)\\ \leq \max_{\forall k,t}\left(\left|a\cdot x_{k,t}^{}\right|\right)+\max_{\forall k,t}\left(\left|\left(1-a\right)\cdot x_{k,t}^{}\right|\right)\\ =a\cdot \max_{\forall k,t}\left(\left|x_{k,t}^{}\right|\right)+\left(1-a\right)\cdot \max_{\forall k,t}\left(\left|y_{k,t}^{}\right|\right)=a\cdot f\left(x\right)+\left(1-a\right)\cdot f\left(y\right). \end{array}$$

From the definition of convex function [25], we can conclude that the custom matrix norm is convex.

## References

[1] Hu Z., Cheng J., Zhang Z., Liang Y. (2018). Performance analysis of collaborative beamforming with outdated CSI for multi-relay spectrum sharing networks. IEEE Trans. Veh. Technol..

[2] Wang C., Haider F., Gao X., You X., Yang Y., Yuan D., Aggoune H.M., Haas H., Fletcher S., Hepsaydir E. (2014). Cellular architecture and key technologies for 5G wireless communication networks. IEEE Commun. Mag..

[3] Chen Y., Lin L., Cao G., Chen Z., Li B. Stable Combinatorial Spectrum Matching. Proceedings of the IEEE Conference on Computer Communications (IEEE INFOCOM 2018).

[4] Imana E.Y., Yang T., Reed J.H. (2017). Suppressing the effects of aliasing and IQ imbalance on multiband spectrum sensing. IEEE Trans. Veh. Technol..

[5] Marcus M., Burtle J., Franca B., Lahjouji A., McNeil N. (2002). Federal Communications Commission Spectrum Policy Task Force. https://www.fcc.gov/sptf/files/EUWGFinalReport.doc.

[6] Yao R., Liu Y., Liu J., Zhao P., Ci S. (2015). Utility-based H.264/SVC video streaming over multi-channel cognitive radio networks. IEEE Trans. Multimed..

[7] Ozger M., Alagoz F., Akan O.B. (2018). Clustering in multi-channel cognitive radio ad hoc and sensor networks. IEEE Commun. Mag..

[8] Kafaie S., Chen Y., Dobre O.A., Ahmed M.H. (2018). Joint inter-flow network coding and opportunistic routing in multi-hop wireless mesh networks: A comprehensive survey. IEEE Commun. Surv. Tutor..

[9] Shu Z., Qian Y., Yang Y., Ahmed M.H. (2016). A cross-layer study for application-aware multi-hop cognitive radio network. Wirel. Commun. Mob. Comput..

[10] Zhao J., Cao G. (2014). Robust topology control in multi-hop cognitive radio networks. IEEE Trans. Mob. Comput..

[11] Wang J., Yue H., Hai L., Fang Y. (2017). Spectrum-aware anypath routing in multi-hop cognitive radio networks. IEEE Trans. Mob. Comput..

[12] Syed A.R., Yau K.A., Qadir J., Mohamad H., Ramli N., Keoh S.L. (2016). Route selection for multi-hop cognitive radio networks using reinforcement learning: An experimental study. IEEE Access.

[13] Sengupta S., Subbalakshmi K.P. (2013). Open research issues in multi-hop cognitive radio networks. IEEE Commun. Mag..

[14] Xu C., Zheng M., Liang W., Yu H., Liang Y. (2017). End-to-end throughput maximization for underlay multi-hop cognitive radio networks with RF energy harvesting. IEEE Trans. Wirel. Commun..

[15] Gallardo G.A., Jakllari G., Canourgues L., Beylot A. (2018). Statistical admission control in multi-hop cognitive radio networks. IEEE/ACM Trans. Netw..

[16] Zheng J., Yang P., Luo J., Liu Q., Yu L. (2016). Per-user throughput analysis for secondary users in multi-hop cognitive radio networks. Comput. Netw..

[17] Tang F., Li J. (2017). Joint rate adaptation, channel assignment and routing to maximize social welfare in multi-hop cognitive radio networks. IEEE Trans. Wirel. Commun..

[18] Li M., Salinas S., Li P., Huang X., Fang Y., Glisic S. (2015). Optimal scheduling for multi-radio multi-channel multi-hop cognitive cellular networks. IEEE Trans. Mob. Comput..

[19] Liu Z. (2019). Resource allocation strategy against selfishness in cognitive radio ad-hoc network based on Stackelberg game. IET Commun..

[20] López O.L.A., Sánchez S.M., Mafra S.B., Fernandez E.M.G., Brante G., Souza R.D. (2018). Power control and relay selection in cognitive radio ad hoc networks using game theory. IEEE Syst. J..

[21] Liu X., Jia M., Na Z., Lu W., Li F. (2017). Multi-modal cooperative spectrum sensing based on dempster-shafer fusion in 5G-based cognitive radio. IEEE Access.

[22] Rardin R.L. (1998). Optimization in Operations Research.

[23] Dinkelbach W. (1967). On nonlinear fractional programming. Manag. Sci..

[24] Papandriopoulos J., Evans J.S. (2009). SCALE: A low-complexity distributed protocol for spectrum balancing in multiuser DSL networks. IEEE Trans. Inf. Theory.

[25] Boyd S., Vandenberghe L. (2004). Convex Optimization.

[26] Arienzo L., Tarchi D. (2015). Statistical modeling of spectrum sensing energy in multi-hop cognitive radio networks. IEEE Signal Process. Lett..

[27] Shrestha A.P., Yoo S. (2018). Optimal resource allocation using support vector machine for wireless power transfer in cognitive radio networks. IEEE Trans. Veh. Technol..

[28] Tu Z., Lu Y. (2004). A robust stochastic genetic algorithm (StGA) for global numerical optimization. IEEE Trans. Evol. Comput..

[29] Ilie D. (2007). Optimization Algorithms with Applications to Unicast QoS Routing in Overlay Networks.

[30] Deb K., Pratap A., Agarwal S., Meyarivan T. (2002). A fast and elitist multiobjective genetic algorithm: NSGA-II. IEEE Trans. Evol. Comput..

